# Supplementary material for: The AMP deaminase of the mollusk Helix pomatia is an unexpected member of the adenosine deaminase-related growth factor (ADGF) family
Source: PLoS One. 2023 Jul 20;18(7):e0286435. doi: 10.1371/journal.pone.0286435 (PMC10358891; doi:10.1371/journal.pone.0286435)
Supplement: Attachment — Submitted filename: Response To Reviewers.docx [file pone.0286435.s004.docx]

Response To Reviewers

Thank you for the reviews of the manuscript entitled “**The AMP deaminase of the mollusk *Helix pomatia* is an unexpected member of the adenosine deaminase-related growth factor (ADGF) family**”*.*

Response to Editor comments:

1. Concerning Style requirements, we found the supplemental S1 Table which was incorrectly named and corrected it. Style was also corrected for author affiliations.
2. We have added captions for supporting information files at end of the manuscript.
3. Four references have been added to the reference list (6,24,25,26). We have reviewed the reference list and ensure it is complete and correct.

Reviewer 1

1. I apologize for failing to notice a key reference was missing, which has now been added (6).
2. As suggested replaced “identity score” with “protein sequence identity”.
3. Added reference 18 as suggested.
4. Added following addressing question about homodimer:

Furthermore, Alphafold2 predicted that HPAMPD is a homodimer, as is the experimentally derived structure for the human ADGF (18). The extensive dimer interactions described for the human ADGF are also present in the predicted homodimer of HPAMPD (S3 Fig.).

5. As suggested added Niedzwicki reference (24) and changed text accordingly. (page 10)

Reviewer 2

1. Fig 6 is indeed blurry in the pdf version of the manuscript, but the link in the pdf downloads the corresponding higher quality Tif file, which has been processed by the PACE diagnostic tool. If the Tif file is a problem, please get back to me.
2. Rechecked and corrected the paper for spaces between words and numbers.
3. We corrected on page 10, “54 ° C” to “54° C”.
4. Corrected line 178 with complete sentence:

The recombinant form of the HPAMPD has a similar spectrum of activity on adenyl substrates as the native enzyme preparation from this study and the enzyme characterized in the 1984 Stankiewicz paper (2) (Table 1).

5. Added 3 sentences near beginning of the first paragraph in Discussion to address comment for line 225.
6. Added clarifying statement to end of first paragraph in Discussion about annotation of ADGFs.
